# Transcriptome of Gonads From High Temperature Induced Sex Reversal During Sex Determination and Differentiation in Chinese Tongue Sole, *Cynoglossus semilaevis*

**DOI:** 10.3389/fgene.2019.01128

**Published:** 2019-11-22

**Authors:** Qian Wang, Kaiqiang Liu, Bo Feng, Zhihua Zhang, Renkai Wang, Lili Tang, Wensheng Li, Qiye Li, Francesc Piferrer, Changwei Shao

**Affiliations:** ^1^Key Lab of Sustainable Development of Marine Fisheries, Ministry of Agriculture, Yellow Sea Fisheries Research Institute, Chinese Academy of Fishery Sciences, Qingdao, China; ^2^Laboratory for Marine Fisheries Science and Food Production Processes, Pilot National Laboratory for Marine Science and Technology (Qingdao), Qingdao, China; ^3^College of Fisheries and Life Science, Shanghai Ocean University, Shanghai, China; ^4^Laizhou Mingbo Aquatic Co., Ltd., Laizhou, China; ^5^BGI-Shenzhen, Shenzhen, China; ^6^Institut de Ciències del Mar (ICM), Consejo Superior de Investigaciones Científicas (CSIC), Barcelona, Spain

**Keywords:** transcriptome, high temperature, sex differentiation, environmental sex reversal, *Cynoglossus semilaevis*

## Abstract

The sex of Chinese tongue sole (*Cynoglossus semilaevis*) is determined by both genetic sex determination (GSD) and environmental sex determination (ESD), making it an ideal model to study the relationship between sex-determination and temperature. In the present study, transcriptomes of undifferentiated gonads from genetic females and males, as well as differentiated gonads from males, females, and pseudomales under high and normal temperature treatments were generated for comparative transcriptomic analysis. A mean of 68.24 M high-quality clean reads was obtained for each library. Differentially expressed genes (DEGs) between different sexes and environmental treatments were identified, revealing that the heat shock protein gene family was involved in the high temperature induced sex reversal. The Gene Ontology (GO) terms and Kyoto Encyclopedia of Genes and Genomes (KEGG) pathways that were enriched in pseudomale and genetic female comparison included neuroactive ligand-receptor interaction, cortisol synthesis and secretion, and steroid hormone biosynthesis. Furthermore, weighted gene co-expression network analyses were conducted on all samples, and two modules were positive correlated with pseudomale under high temperature. An illustrated protein-protein interaction map of the module identified a hub gene, *hsc70*. These findings provide insights into the genetic network that is involved in sex determination and sexual differentiation, and improve our understanding of genes involved in sex reversal under high temperature.

## Introduction

Sexually dimorphic traits play a dominant role in evolution and behavior of animals, and genes regulating sex-determination and sexual development have long been of central interest in developmental biology. In vertebrates, sex determination mechanisms are broadly divided into two major categories: genetic sex determination (GSD) and environmental sex determination (ESD) (Marshall Graves, 2008). In GSD, the primary sex is determined during fertilization and regulated by heritable genetic components (Budd et al., 2015). In contrast, organisms possess ESD have undifferentiated gonads until they reach a sensitive period perceiving the environmental factors to determine the sex (Shao et al., 2014). Interestingly, some GSD species, mainly fish and reptiles, can change their primary sex without changing the genotype when exposed to environmental factors, a process known as environmental sex reversal (ESR) (Sarre et al., 2011; Shao et al., 2014; Holleley et al., 2015). Temperature is the most broadly studied factor that induces ESR. For example, Nile tilapia (*Oreochromis niloticus*) exposed to elevated temperature can override genetic sex determination, and the females can be reversed to produce pseudomales (Baroiller et al., 2009). Blue tilapia (*Oreochromis aureus*), Japanese flounder (*Paralichthys olivaceus*), European sea bass (*Dicentrarchus labrax*), turbot (*Scophthalmus maximus*) and Atlantic silverside (*Menidia menidia*) also undergo temperature induced ESR (Conover and Fleisher, 1986; Desprez and Mélard, 1998; Yamaguchi et al., 2007; Diaz and Piferrer, 2015; Robledo et al., 2015; Diaz and Piferrer, 2017).

Previous studies have found that epigenetic changes are involved in regulating ESR. In the European sea bass and Nile tilapia, juveniles exposed to high temperatures show increased methylation in the *cyp19a1a* promoter, which correlated to the masculinization of these individuals (Navarro-Martin et al., 2011; Wang et al., 2017). In red-eared slider turtle (*Trachemys scripta elegans*) (ESD), high temperature lead KDM6B, the histone H3 lysine 27 demethylase, to promote the transcription of the male sex-determining gene *dmrt1*. Therefore, a direct genetic link between epigenetic mechanisms and temperature-dependent sex determination has been shown in a turtle species (Ge et al., 2018).

Development of sexual traits is a complex process that involves a network of genes. Genes related to sex-determination (e.g. *cyp19a1a* and *amh*) and epigenetic regulators appear to be downstream factors in the ESR related pathways. Therefore, the identification of more upstream genes responding to the change in temperature is critical to further our understanding of ESR. Transcriptomic analyses can serve as a powerful tool to identify potential upstream regulators. The gonad transcriptome of Nile tilapia revealed numerous genes involved in temperature dependent ESR (Sun et al., 2018). In sea bass and pejerrey (*Odontesthes bonariensis*), transcriptomes of the developing gonads identified pathways involved in sex differentiation affected by temperature (Fernandino et al., 2011; Diaz and Piferrer, 2015). These reports were either in mature fish or did not test for fish that underwent ESR. Thus, genes involved in early development and temperature-induced ESR remains elusive.

Chinese tongue sole (*Cynoglossus semilaevis*) is an economically important marine flatfish with ZZ/ZW sex chromosome system (GSD) and the genome has been sequenced (Chen et al., 2014). Genotypic females of the Chinese tongue sole can be transformed into pseudomales when exposed to high temperatures during the crucial stage of sex differentiation, and epigenetic regulation is important during this process (Chen et al., 2014; Shao et al., 2014). Therefore, *C*. *semilaevis* is an ideal model to illuminate the relationship between sex-determination and temperature during ESR. Previous studies identified genes related to sexual dimorphism through transcriptome analysis of adult *C*. *semilaevis* (Guo et al., 2016; Wang et al., 2018; Liu et al., 2019). Gene expression patterns in the gonads of *C*. *semilaevis* before and after ESR remain unclear, partly due to the difficulty in distinguishing pseudomales at early stage. Excitedly, recent studies have uncovered sex determining gene *dmrt1*, and new single nucleotide polymorphism (SNP) loci associated with pseudomales, allowing for the detection of male, female, and pseudomale *C*. *semilaevis* (Cui et al., 2017; Jiang and Li, 2017; Cui et al., 2018).

In the present study, transcriptomic analysis was conducted on undifferentiated gonads from genetic females and males, plus differentiated gonads from males, females, and pseudomales under high temperature and normal temperature treatment. A large number of differentially expressed genes (DEGs) between female, male, and pseudomales under high and normal temperature were obtained. DEGs were functionally analyzed *via* GO term and KEGG pathway enrichment. The heat shock protein (*hsp*) gene family and several pathways, such as steroid hormone biosynthesis, cortisol synthesis and secretion, and neuroactive ligand-receptor interaction, were differentially expressed. Weighted gene co-expression network analysis (WGCNA) was employed to identify pseudomale-related genetic modules and *hsc70* was identified as hub gene regulates the formation of pseudomales. These results provide new insights into the genes involved in sex determination and ESR in *C*. *semilaevis*.

## Materials and Methods

### Fish Culture and High Temperature Induction

*C. semilaevis* were kept in Laizhou Mingbo Aquatic Co., Ltd., in Yantai, China. Approximately 300 larvae from the same family were fed twice a day with commercial pellets and were reared in filtered seawater at 22°C. For the undifferentiated group (30 dpf_F and 30dpf_M), twenty larvae were sampled at 30-day post fertilization (dpf). The remaining larvae were divided into two equal groups and cultured in two 300 L tanks. The control group was cultured at 22°C (C22_F, C22_M, C22_P), and the high temperature group was cultured at 28°C (C28_F, C28_M, C28_P) until 3-month post fertilization (mpf) when sex differentiation was completed. Gonads were collected from the 30 dpf and 3 mpf *C. semilaevis* individuals for RNA extraction and immediately stored in liquid nitrogen. The remaining part of each fish was stored in ethanol to identify the genetic sex.

### Genetic Sex Identification of *C. Semilaevis*

Genomic DNA was extracted from small pieces of tail fin using the phenol-chloroform method, and genetic sex was identified by polymerase chain reaction (PCR) against sex-specific simple sequence repeat (SSR) marker with the primers described previously (Liu et al., 2014). Pseudomales at 3 mpf were identified by PCR to detect the two SNP loci associated with sex reversal as previously described (Jiang and Li, 2017; Cui et al., 2018). The relative expression level of *dmrt1* was measured to further confirm male, female, and pseudomale individuals by real-time quantitative reverse transcription PCR (qRT-PCR) (Cui et al., 2017).

### Total RNA Extraction and RNA Sequencing

Total RNA was isolated from each gonad sample using the Trizol reagent (Invitrogen, USA) according to the manufacturer’s instructions. The qualities of the RNA samples were measured with the Agilent 2100RNA 6000 Nano kit (Agilent, USA). Three biological replicate samples for each group were generated. Thus, 24 samples of 30 dpf_F1-3, 30 dpf_M1-3, C22_F1-3, C22_M1-3, C22_P1-3, C28_F1-3, C28_M1-3, and C28_P1-3 were acquired. RNA sequencing was conducted with a BGISEQ-500 by PTM Biolabs (Hangzhou, China).

### Transcriptome Quality Control and Gene Annotation

The sequencing reads were filtered using the SOAPnuke v1.4.0 (Chen et al., 2018) and trimmomatic (Bolger et al., 2014) software using default parameters to remove adapters, low quality reads with more than 5% unknown nucleotides, and reads with more than 20% low-quality bases (Q value <10). The filtered clean data were subsequently mapped to the *C. semilaevis* genome (NCBI Cse_v1.0) *via* HISAT2 v2.1.0 (Kim et al., 2015) and aligned with the reference transcript sequence using Bowtie2 v2.2.5 (Langmead and Salzberg, 2012) for removing rRNA sequences and annotating genes.

### Analysis of Differentially Expressed Genes

Abundances of transcripts were quantified by RNA-Seq by Expectation Maximization (RSEM) (Li and Dewey, 2011), and gene expression level was further normalized using the Fragments Per Kilobase Million (FPKM) method to eliminate the influence of different gene lengths and amount of sequencing data on the calculation of gene expression. Differential expressed genes (DEGs) were identified using DEGseq and DEseq2 (Wang et al., 2010; Love et al., 2014). Transcripts with a fold change ≥2 and adjusted *p* (*q* value) < 0.001 were considered to be significant DEGs. DEGs were then subjected to an enrichment analysis for GO function and KEGG pathways using Phyper in R (Johnson et al, 1992), with *p* value < 0.05 as threshold. The cluster analysis of DEGs was processed using Pheatmap in R (https://CRAN.R-project.org/package=pheatmap). 

### Gene Co-Expression Network Analysis

To further understand the relationships between DEGs, a network analysis based on gene-to-gene correlations was performed using the R package WGCNA V1.48 (Langfelder and Horvath, 2008). Expression correlation coefficients of genes were calculated to search a suitable soft threshold to build gene networks using a scale-free topology model. Subsequently, gene modules with similar expression patterns were identified based on the resulting gene cluster dendrogram and using the dynamic tree cut method (GeneFrac Threshold 0.5). GO term and KEGG pathway enrichment analyses of the annotated genes were performed for each module and *p* value < 0.05 was defined as the threshold for significance. The protein–protein interaction network was predicted using DIAMOND (Buchfink et al., 2015) to map the genes to the STRING database, and score ≥300 was set as the threshold.

### Validation of RNA-Seq Data by qRT-PCR

Nine candidate genes related to high temperature induced sex-reversal from the female, pseudomale, and male between the heat versus control temperature were validated with qRT-PCR. Primers (**Table S1**) were designed based on sequences from the National Center for Biotechnology Information (NCBI) database. *β-actin* gene was used as the internal control (Wang et al., 2018). One microgram total RNAs for high-throughput transcriptome sequencing was reverse transcribed into cDNA with the PrimeScript^™^ RT reagent Kit with gDNA Eraser (Takara, Japan). Then, qRT-PCR was performed using QuantiNova^™^ SYBR Green PCR Kit (Qiagen, Germany) in 20-µl reactions, containing 10 µl 2 × SYBR Green PCR Master Mix, 2 µl QN ROX Reference Dye, 0.7 µM forward primer, 0.7 µM reverse primer, and 1 µl cDNA. The cycling program was carried out at 95°C for 2 min, followed by 40 cycles of 95°C for 5 s and 60°C for 10 s; this was followed by a melting curve analysis in an ABI StepOnePlus Real-Time PCR system (Applied Biosystems, USA). Reactions were performed in triplicate. The relative expression fold changes of nine genes in female, pseudomale, and male gonads under normal and high temperature were analyzed using the 2^−ΔΔCt^ method.

## Results

### Phenotypic and Genetic Sex Identification of *C. Semilaevis*

The average weight of larvae sampled at 30 dpf was 0.080 ± 0.004 g, with the average length of 2.513 ± 0.041 cm. At 3 mpf, the average weight of normal temperature group was 1.312 ± 0.080 g, with the average length of 5.689 ± 0.136 cm. The average weight of high temperature group was 1.916 ± 0.127 g, and the average length of 6.911 ± 0.206 cm. The genetic sex of 30 dpf and 3 mpf female and male were distinguished using PCR of SSR marker. The genetic male had Z-specific derived band, and the genetic female had both W-specific and Z-specific derived bands. The genetic females that carried the T allele of Cyn_Z_6676874 and the A allele of Cyn_Z_8564889 may change into pseudomales. In addition, the relative expression of *dmrt1* was high in males, low in females, and moderate in pseudomales (**Figure S1**).

### Gonadal Transcriptome of *C. Semilaevis*

A total of 24 libraries, named C22_F 1-3, C22_M 1-3, C22_P 1-3, C28_F 1-3, C28_M 1-3, C28_P 1-3, 30dpf_F 1-3, 30dpf_M 1-3, were constructed using total RNA from gonads. A mean of 68.24 M filtered clean reads with a Q20 above 96.81% was obtained from each library (**Table S2**). Of the clean reads, 87.82–90.80% mapped to the *C. semilaevis* genome (NCBI Cse_v1.0) (**Table S3**), and a total of 25,022 known genes were identified in the transcriptome based on the *C. semilaevis* genome. The transcriptome data has been uploaded to NCBI Sequence Read Archive (SRA). The accession number was PRJNA576366.

### Analysis of Differentially Expressed Genes

**Figure 1:**
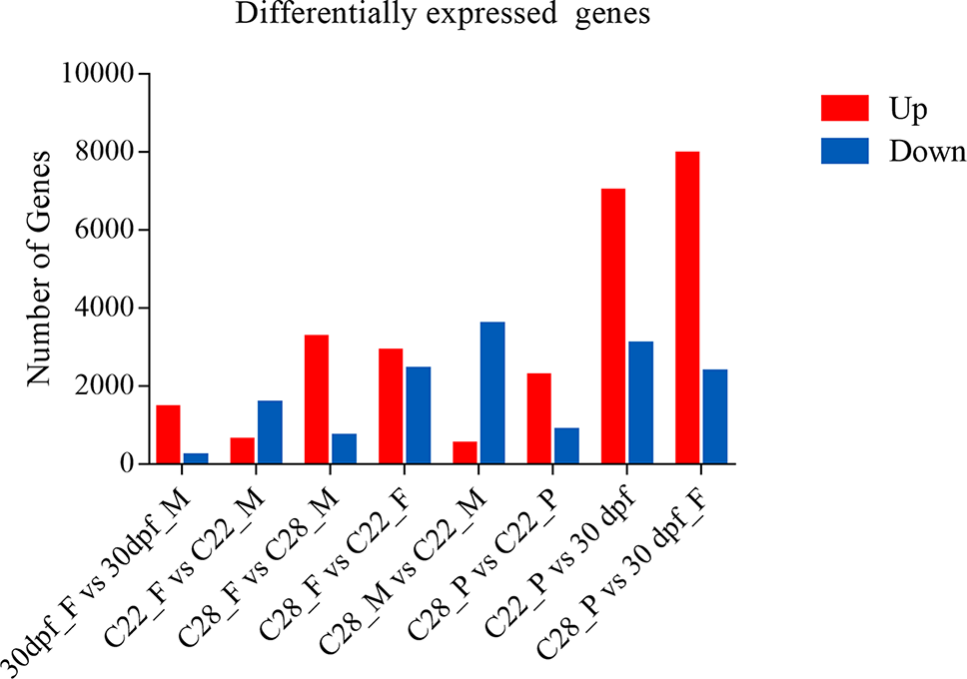
Number of differentially expressed genes. DEGs identified from each comparison groups. Red and blue indicate up-regulated genes and down-regulated genes, respectively.

Using an adjusted *p* value of less than 0.001 and an absolute fold change greater than 2 as the threshold, over 1500 DEGs were obtained for each comparison, and the number of DEGs were highly variable, depending on the sex and environmental temperature treatment (**Figure 1**).

### GO and KEGG Enrichment Analyses of DEGs between Females and Males

To explore differences in gene expression between genetic females and males before sex differentiation, GO and KEGG enrichment analyses of DEGs between 30dpf_F and 30dpf_M was conducted. In the gonadal primordium region, 13 GO terms were significantly enriched with *q* < 0.05. The enriched GO terms were mainly related to muscle development, including muscle organ development, striated muscle cell development and differentiation (**Figure S2A**). Subsequently, KEGG enrichment analysis revealed that 17 pathways were significantly enriched in the gonadal primordium region (*q* < 0.05), including complement and coagulation cascades, calcium signaling pathway, and apelin signaling pathway (**Figure S2B**).

Genes that were differentially expressed in phenotypic females and males after sex differentiation were evaluated by identifying DEGs in C22_F vs C22_M. There were 10 GO significantly enriched (*q* < 0.05). Including DNA integration, microtubule-based process, DNA conformation change, nucleosome assembly, and protein-DNA complex assembly (**Figure S2C**). KEGG enrichment analysis identified 32 significantly enriched pathways in the differentiated gonads (*p* < 0.05). Among these, nine pathways were significantly enriched (*q* < 0.05), including pancreatic secretion, calcium signaling pathway, circadian entrainment, and oocyte meiosis (**Figure S2D**).

Gene expression changes in individuals exposed to high temperature conditions were identified by comparing C28_F vs C28_M. There were 221 significantly enriched GO terms (*p* < 0.05), including DNA binding, aminoglycan metabolic process, ion gated channel activity, DNA metabolic process, and temperature homeostasis (**Figure S2E** and **Table S4**). In addition, there were 20 enriched KEGG pathways (*q* < 0.05), including neuroactive ligand-receptor interaction, cell cycle, p53 signaling pathway, and ovarian steroidogenesis (**Figure S2F**). In addition, circadian entrainment, oocyte meiosis, and calcium signaling pathway were enriched, and these three pathways were also enriched in the in the C22_F vs C22_M comparison.

**Figure 2:**
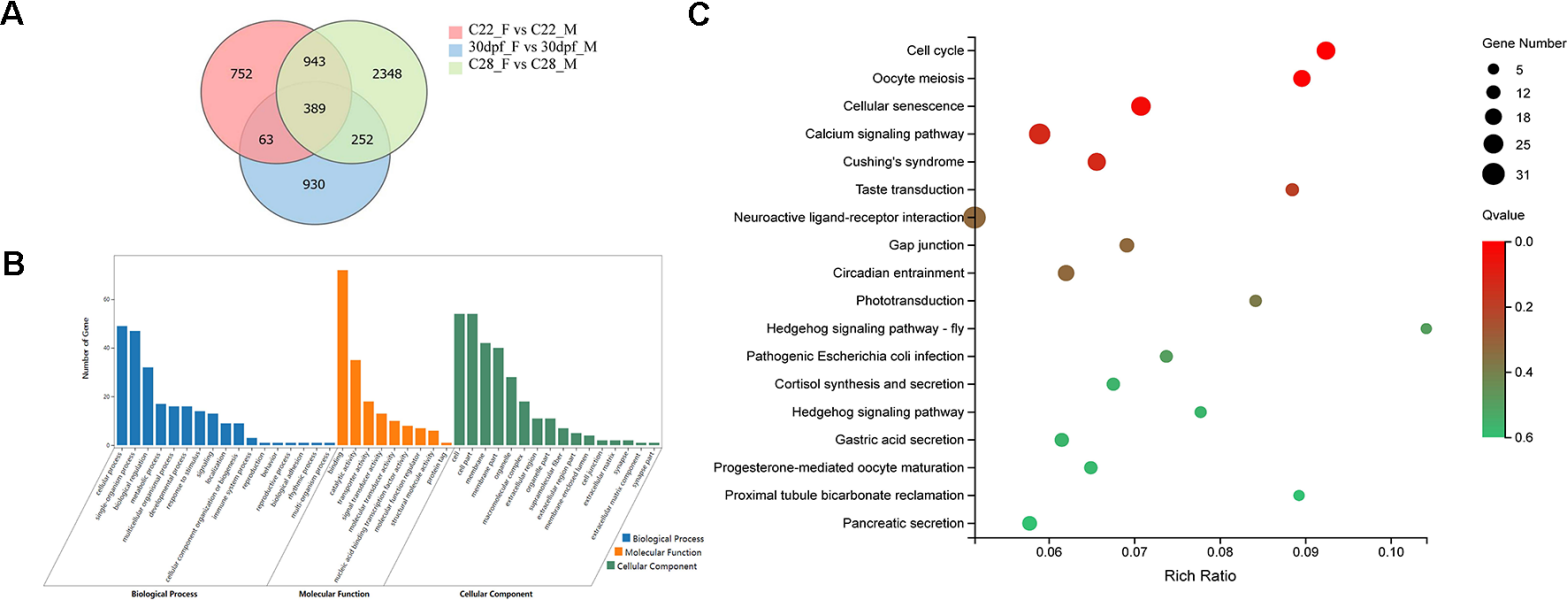
Analysis of DEGs between females and males. **(A)** Number of DEGs between females and males shown by Venn diagram. **(B)** GO classifications of 943 DEGs belong to C22_F vs C22_M and C28_F vs C28_M excluding 30dpf_F vs 30dpf_M. **(C)** Enriched KEGG pathways of the 943 DEGs (*p* < 0.05).

DEGs that were identified from the 30dpf_F vs 30dpf_M comparison were mainly enriched in genes involved in muscle development, therefore we focused on the remaining shared 943 DEGs that were identified from the C22_F vs C22_M and C28_F vs C28_M comparisons, which included sex-related genes, such as *figla*, *foxl2*, *amh*, and *dmrt1*. DEGs were further analyzed using GO annotations to identify their potential functions. In biological process, cellular process, single-organism process, and biological regulation were the most abundant GO function items, while binding, catalytic activity, and transporter activity were most abundant in molecular function. In cellular component, GO terms for cell, cell part, and membrane were enriched. In KEGG enrichment analysis, 18 KEGG pathways were significantly enriched (*p* < 0.05), including cell cycle, oocyte meiosis, hedgehog signaling pathway, calcium signaling pathway, neuroactive ligand-receptor interaction, and pancreatic secretion (**Figure 2**).

### The GO and KEGG Enrichment Analysis of DEGs Between Fish Exposed to High and Normal Temperatures

**Figure 3:**
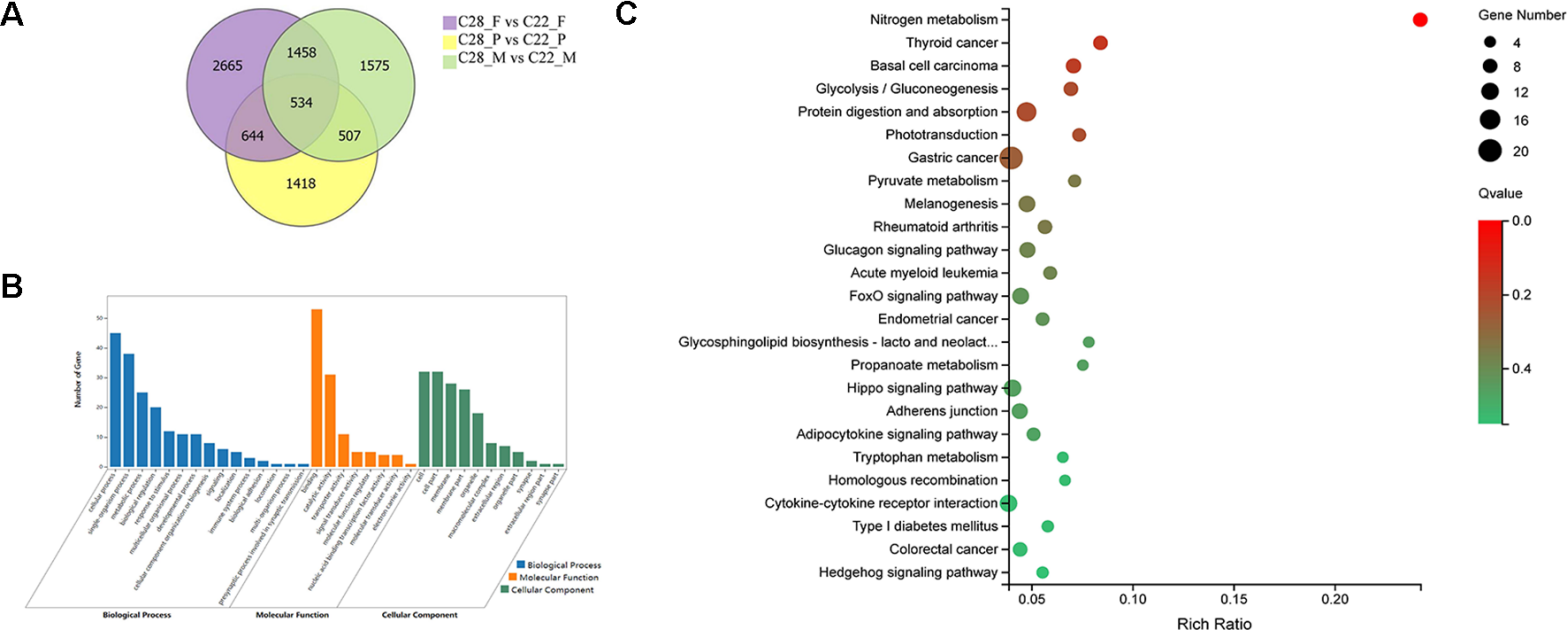
Analysis of DEGs in females, males, and pseudomales between high temperature and normal temperature. **(A)** Number of DEGs between high temperature and normal temperature shown by Venn diagram. **(B)** GO classifications of 534 DEGs in females, males and pseudomales between high temperature and normal temperature. **(C)** Enriched KEGG pathways of the 534 DEGs (*p* < 0.05).

To explore the gonadal-specific effects of temperature, DEGs were identified for females, males and pseudomales under high and normal temperature treatments. There were 534 DEGs shared in females, males and pseudomales when exposed to high temperature. DEGs were most enriched in the biological process functional items including cellular process, single-organism process, and metabolic process. Binding, catalytic activity, and transporter activity were most abundant in molecular function. In cellular component, cell, cell part, and membrane, were enriched. In KEGG enrichment analysis, 25 KEGG pathways were significantly enriched (*p* < 0.05), including nitrogen metabolism, glucagon signaling pathway, phototransduction, adipocytokine signaling pathway, FoxO signaling pathway, and hippo signaling pathway (**Figure 3**).

### Analysis of DEGs Between Pseudomale Formed Under High Temperature and Normal Temperature

**Figure 4:**
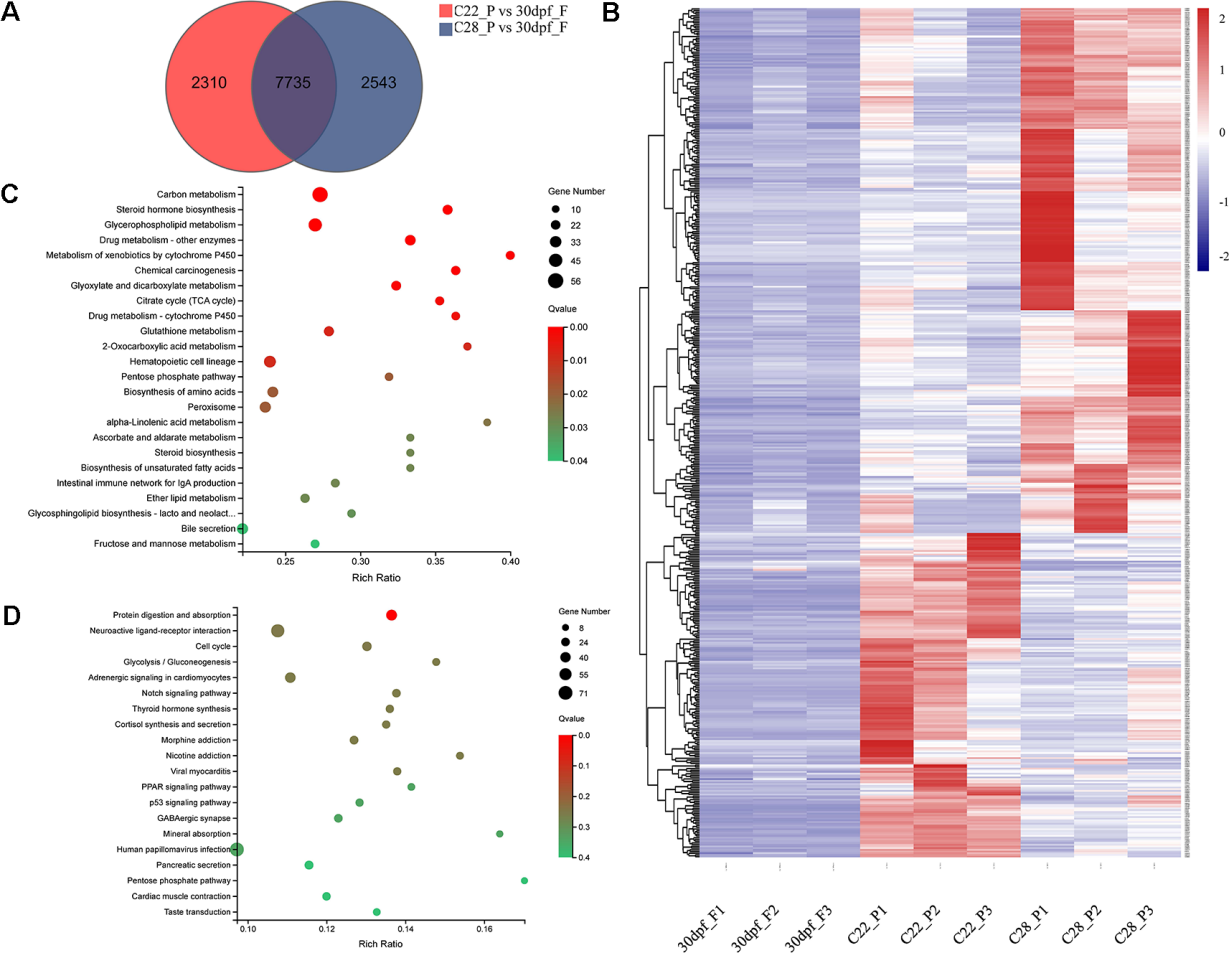
Gene expression patterns in pseudomale formed under high temperature and normal temperature. **(A)** Number of DEGs shown by Venn diagram. **(B)** Heatmaps showing the expression of DEGs found both in C22_P vs 30dpf_F and C28_P vs 30dpf_F (red: higher expression, blue: lower expression). **(C)** Enriched KEGG pathways of DEGs up-regulated in C28_P compared to C22_P (*q* < 0.05). **(D)** Enriched KEGG pathways of DEGs down-regulated in C28_P compared to C22_P (*q* < 0.05), graph showing top 20 significant terms.

Pseudomales develop from genetic females. To identify the changes in gene expression in pseudomales under high and normal temperature conditions, 7735 DEGs in both C22_P vs 30dpf_F and C28_P vs 30dpf_F comparisons were identified (**Figure 4A**). These DEGs were further screened by their relative expression pattern in C22_P and C28_P, and then divided into two categories (**Figure 4B** and **Table S5**). In the first category, heat shock protein family, including *hspa13* (heat shock protein family A (Hsp70) member 13), *hspa5* (heat shock protein family A (Hsp70) member 5), *hsp90b1* (heat shock protein 90 beta family member 1), *hspd1* (heat shock protein family D (Hsp60) member 1), and *hspb9* (heat shock protein family B (small) member 9) were up-regulated in C28_P compared to C22_P. The male-related genes *dmrt1* and *sox6* were also up-regulated in C28_P compared to C22_P. Other genes that were up-regulated in the high temperature treated pseudomales included the *vasopressin V2 receptor* (LOC103384583), which is involved in signal transduction, the *bag3* and *BAG family molecular chaperone regulator 4-like* (LOC103398065) which were co-chaperones for HSP70 and HSC70 chaperone proteins, and *cyp11b-like*, which is responsible for glucocorticoid and mineralocorticoid biosynthesis. KEGG enrichment analysis of this group of genes found 24 enriched pathways (*q* < 0.05). These pathways included steroid hormone biosynthesis, metabolism of xenobiotics by cytochrome P450, and carbon metabolism (**Figure 4C**). The second category included *hsd17b3-like* (testosterone 17-beta-dehydrogenase 3-like), *fshr* (follicle stimulating hormone receptor), *aromatase-like*, female-related *foxl2* and *wnt5b*, and 11*β*-hydroxysteroid dehydrogenases *hsd11b1-like*, which were down-regulated in C28_P compared to C22_P. These DEGs had 30 significantly enriched KEGG pathways (*q* < 0.05), including neuroactive ligand-receptor interaction, cortisol synthesis and secretion, progesterone-mediated oocyte maturation, and estrogen signaling pathway (**Figure 4D** and **Table S6**)

### Weighted Gene Correlation Network Analysis and Functional Annotation of Pseudomale-Related Modules

**Figure 5:**
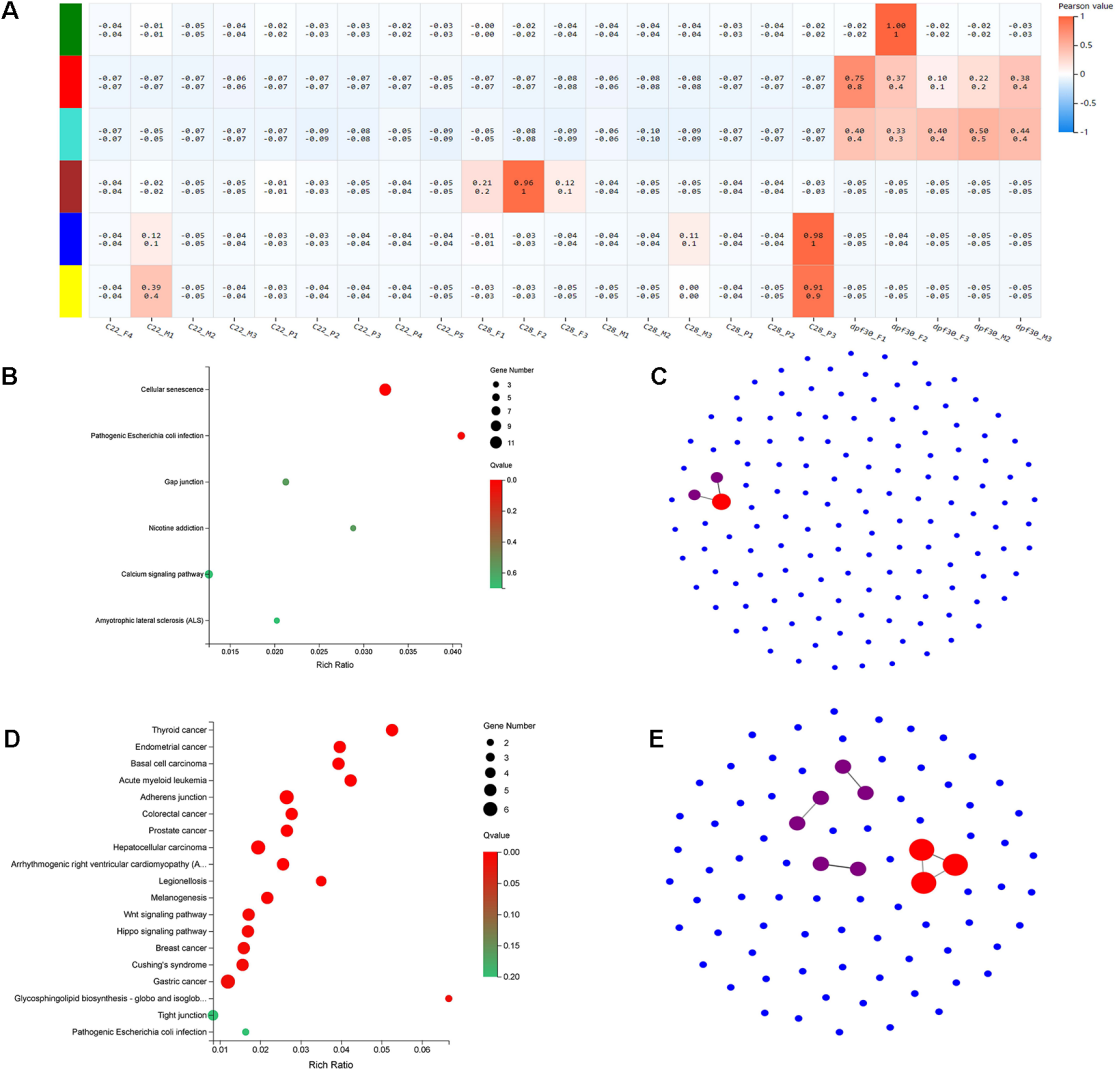
WGCNA and functional analysis of pseudomale-related modules. **(A)** The relationship between 6 modules across all samples by weighted gene coexpression network analysis (WGCNA). The color bar indicates correlation value from low (blue) to high (red). **(B**, **D)** Enriched KEGG pathways of genes in the blue module **(B)** and yellow module **(D)** (*q* < 0.05). **(C**, **E)** The network relationship in the blue module **(C)** and yellow module **(E)**.

To identify the genetic networks involved in the formation of pseudomales in *C. semilaevis*, a weighted gene coexpression network analysis (WGCNA) was conducted on all samples. Based on the expression values of each gene, we ultimately obtained six modules, and genes within each module had highly interconnected expression patterns (**Figure 5A**). According to the correlation coefficients between the module and the samples, we found that the blue and yellow modules showed the highest and significant correlation with the pseudomale gonad (Pearson value >0.9). We performed KEGG enrichment analysis on the two modules, and found that the blue module had 140 genes that were significantly enriched in pathways related to signal transduction, including cellular senescence, gap junction, and calcium signaling pathway. This suggests that genes in the blue module are likely involved in sensing external high temperature and activating signals (**Figure 5B**). In the yellow module, 83 genes were significantly enriched in 19 pathways (*p* < 0.05). The enriched KEGGs included Wnt signaling pathway, hippo signaling pathway, glycosphingolipid biosynthesis-globo and isoglobo series, adherens junction, and melanogenesis (**Figure 5D**). The co-expression network of the blue and yellow modules was further explored by protein-protein interaction analysis. The hub gene in the blue module was leucine-rich repeat-containing protein 18 (**Figure 5C**). The hub genes in the yellow module were heat shock cognate 70 kDa protein-like (XP_008307673.1), elongation factor 1-alpha 1 (XP_020457373.1), and eukaryotic initiation factor 4A-II-like (XP_008332345.1) (**Figure 5E**).

### Validation of Transcriptome Sequencing by qRT-PCR

To validate the transcriptome sequencing data, the fold changes in 9 genes including *figla*, *foxl2*, *wnt5b*, *hspa13*, *hsc70-like*, *hsd11b1-like*, *hsd17b3-like*, *aromatase-like*, and *cyp11b-like* were measured using qRT-PCR. Similar up- or down-regulation patterns of these genes were observed in qRT-PCR and RNA-seq results (**Figure S3**), although few genes showed differentiation between the values. These differentiations may be due to the different calculated methods used between RNA-seq and qPCR. In general, the validation analysis by qRT-PCR indicated the accuracy and reliability of the RNA-seq results.

## Discussion

In the last decade, effort has been made to identify the molecular mechanism that underlies ESR in fish. High throughput sequencing, microarray profiles and genome-wide methylation analysis in zebrafish, European sea bass, and Nile tilapia have identified the role of epigenetic inheritance in ESR (Diaz and Piferrer, 2015; Sun et al., 2016; Ribas et al., 2017). A recent study in *T. scripta* found that epigenetic factors activate sex-related genes during male sex determination under high temperature (Ge et al., 2018). However, the relationship between high temperature and the epigenetic mechanisms, and the factors that convert external high temperature signals into internal signals to then alter the sex of genetic females to phenotypic males largely remains unclear.

To further identify genes involved in ESR, we exposed *C*. *semilaevis* to high temperature conditions during early sex differentiation from 30 dpf to 3 mpf. We focused on identifying gene expression changes in the gonad of males, females and pseudomales at the undifferentiated and post-differentiated stages using transcriptomic analysis. Before sex differentiation, genetic males and females showed differences in genes related to muscle development, suggesting that the sex-related dimorphism in *C*. *semilaevis* may be traced back to the pre-sex differentiation stage. After sex differentiation, females had up-regulation of female-specific genes *figla* and *foxl2* and down-regulation of male-specific genes *dmrt1* and *amh*, both in normal and high temperature conditions. DEGs between female and male were enriched in KEGG pathways cell cycle, oocyte meiosis, hedgehog signaling pathway, calcium signaling pathway, neuroactive ligand-receptor interaction, and pancreatic secretion. In the previous study of adult gonads of *C*. *semilaevis*, DEGs between sexes were also enriched in KEGG pathways included cell cycle, oocyte meiosis, and calcium signaling, whereas neuroactive ligand-receptor interaction and pancreatic secretion pathways were not enriched (Wang et al., 2018). Thus, these two pathways may be important during early sex differentiation. When comparing gonads of fish exposed to normal or high temperatures, 534 DEGs were found in all sexes of *C*. *semilaevis*, suggesting that there is likely a common genetic mechanism to cope with exposure to high temperature. These DEGs were enriched in the KEGG pathways nitrogen metabolism, glucagon signaling pathway, protein digestion and absorption, phototransduction, adipocytokine signaling pathway, and FoxO signaling pathway. Therefore, exposure to high temperature influenced metabolism, signal transduction, cell growth and proliferation. The results were consistent with previous studies in *Oncorhynchus mykiss gairdneri* and *Acanthochromis polyacanthus* (Narum and Campbell, 2015; Veilleux et al., 2015).

In order to understand the effect of high temperature on the formation of pseudomales during sex differentiation, we compared the gene expression profiles of 30 dpf genetic females and 3 mpf pseudomales under high or normal temperature treatment. After screening by their relative expression pattern in C22_P and C28_P, the DEGs were divided into two categories: (1) 304 DEGs that were up-regulated in high temperature compared to normal temperature, and (2) 188 DEGs that were down-regulated in high temperature compared to normal temperature. The first category included several members of the heat shock protein family, including *hsp70* and *hsp90* sub-types. Expressions of HSP70 and HSP90 are known to change in response to changes in water temperature in several species (Palmisano et al., 2000; Kazumi, 2002; Currie SMoyes and Tufts, 2010; Fan et al., 2014). In addition, *hsp70* is associated with gonad development in tilapia, where it is highly expressed in the testis during gonad differentiation, along with *sox9a*, *gata4*, and *lhx6* (Tao et al., 2018). Thus, Hsp70 is a candidate sensor for high temperatures during sex differentiation for future studies. The second gene category contained *hsd17b3-like*, which was down-regulated under high-temperature treatment. *hsd17b3* is involved in androgen metabolism through oxidation-reduction reaction (Carre et al., 1993). Reduced expression of *hsd17b3* may facilitate the accumulation of testosterone, thus promoting the formation of pseudomales. These DEGs of the two categories were enriched in several KEGG pathways, including neuroactive ligand-receptor interaction, cortisol synthesis and secretion, and steroid hormone biosynthesis. Within the neuroactive ligand-receptor interaction pathway, the vasopressin receptor gene [*vasopressin V2 receptor* (LOC103384583)] was significantly up-regulated in the pseudomale under high temperature, while *fshr* (follicle stimulating hormone receptor) was significantly down-regulated. Vasopressin is produced and released by the pituitary gland, and mediates peripheral and central physiological functions that are important for osmoregulation and reproduction. Receptors of vasopressin are up-regulated in the *Thalassoma bifasciatum* testis (Liu et al., 2015; Zimmermann-Peruzatto et al., 2015). *fshr* is expressed in the theca and granulosa cells of the ovary, and sertoli cells in the testis. It belongs to the G-protein-coupled membrane receptors superfamily and regulate gametogenesis and production of sexual steroids in teleost gonads (Shinoda et al., 2010). The neuroactive ligand-receptor interaction pathway was also enriched in *Gymnocypris przewalskii* brain under high temperature, where neuroactive ligand-receptor genes exhibited sex- and tissue-specific expression patterns (Tian et al., 2019). The expression of these receptors in the gonad is likely induced by neuropeptides through the hypothalamic-pituitary-gonadal (HPG) axis, which then switches on the expression of genes involved in steroid hormone synthesis.

The steroid hormone biosynthesis pathway plays a critical role in sex differentiation across vertebrates (Devlin and Nagahama, 2002). In addition to *hsd17b3-like* (LOC103398902), *aromatase-like* (LOC103378914) was also found in the steroid hormone biosynthesis pathway. In teleosts, aromatase activity is correlated with sex steroid levels, converting testosterone into estrogen and thus may play a role in homeostasis of the accumulated androgen.

Cortisol is a well-known hormone that is associated with stress and regulates many physiological processes. Cortisol levels are elevated in fish exposed to high temperature or high-density conditions in tilapia, zebrafish, and brook trout (*Salvelinus fontinalis*) (Delaney et al., 2005; Chadwick et al., 2015; Nadirah et al., 2017; Valdivieso et al., 2019). In addition, cortisol is related to masculinization in some fish species such as pejerrey, medaka and the Japanese flounder (Hattori et al., 2009; Hayashi et al., 2010; Yamaguchi et al., 2010). In our study, the cortisol synthesis and secretion pathway was enriched in pseudomales under the high-temperature treatment. *cyp11b-like* was up-regulated and *hsd11b1-like* was down-regulated in C28_P compared to C22_P. CYP11B can catalyze the conversion of testosterone to 11*β*-hydroxytestosterone (precursor of 11-ketotestosterone, 11-KT), a major androgen in fish that promotes spermatogenesis (Nadirah et al., 2017). The down-regulation of 11*β*-hydroxysteroid dehydrogenases *hsd11b1-like* can indicate a shift from cortisone-cortisol regeneration (Morgan et al., 2014). These expression patterns suggested that cortisol might cross-talk with steroidogenesis to mediate sex reversal under high temperature, which coincide with the study in pejerrey and medaka (Fernandino et al., 2012; Fermandino et al., 2013; Castañeda Cortés et al., 2019).

WGCNA analysis gave rise to two modules, although found in one biological replicate, correlated to pseudomale formation under high temperature. These two modules were enriched in KEGG pathways that were involved in signal transduction and cell developmental processes. In particular, protein-protein network analysis of the yellow module in WGCNA revealed *hsc70-like* (heat shock cognate 70 kDa protein-like) as the hub gene for pseudomale formation. HSC70 is a regulator of HSF1 (heat-shock factor 1), which plays an essential role in mediating the appropriate cellular response to physiological stresses (Ahn et al., 2005). In humans, *hsc70* is also a mediator of the CaM-dependent nuclear import of male-specific SRY (Kaur et al., 2013). A previous study showed that heat shock protein 70 kDa (Hsp70/Hsc70) family proteins interact with steroid hormone receptors through BAG (Bcl-2 associated athanogene), and that Bag1L can enhance the transcriptional activity of the androgen receptor (Knee et al., 2001). In our transcriptome data, *bag3* (the Bcl2 associated athanogene 3) and transcript LOC103398065 (BAG family molecular chaperone regulator 4-like) were up-regulated in C28_P compared to C22_P, indicating the linkage between of the heat shock protein family and steroid hormones.

Based on these findings, we propose a mechanism for how the heat signal causes sex reversal. Members of heat shock protein family may detect external high temperature. Then a series of processes including neuroactive ligand-receptor interaction pathway, steroid hormone biosynthesis pathway, and cortisol synthesis and secretion pathway were activated. This leads to changes in the relative amount of sex steroid hormones (androgen and estrogen), promoting the development of pseudomales in *C*. *semilaevis*.

The present study provides important insights into ESR induced pseudomale formation in *C. semilaevis*. Future studies incorporating detailed functional survey of these networks and hub genes will further improve our understanding of sex reversal under external high temperature in fish.

## Data Availability Statement

The transcriptome data used in this study has been uploaded to NCBI Sequence Read Archive (SRA) with accession number PRJNA576366.

## Ethics Statement

The collection and handling of C. semilaevis were approved by the Animal Care and Use Committee at the Chinese Academy of Fishery Sciences, and all the experimental procedures were performed in accordance with the guidelines for the Care and Use of Laboratory Animals at the Chinese Academy of Fishery Sciences.

## Author Contributions

QW analyzed the transcriptome sequencing data and wrote the manuscript. KL and WL reared the fish. BF and ZZ completed fish sampling and SNP locus sequencing. RW finished the qRT-PCR of *dmrt1* gene. LT participated in the fish sampling. QL and FP revised the manuscript. CS was in charge of the guidance and revision of this manuscript.

## Funding

This work was supported by National Nature Science Foundation of China (31722058, 31802275, and 31472269), National Key R&D Program of China (2018YFD0900301), the AoShan Talents Cultivation Program Supported by Qingdao National Laboratory for Marine Science and Technology (2017ASTCP-ES06), the Taishan Scholar Project Fund of Shandong of China to CS, the National Ten-Thousands Talents Special Support Program to CS, and the International Scientific Partnership Program ISPP at King Saud University (No. 0050).

## Conflict of Interest

Author WL was employed by company Laizhou Mingbo Aquatic Co., Ltd. (Laizhou, China). The remaining authors declare that the research was conducted in the absence of any commercial or financial relationships that could be construed as a potential conflict of interest.

## Supplementary Material

Table S1 qRT-PCR primers used for validating RNA-seq data.

Table S2 Reads filtered information of each library.

Table S3 Reads mapping information from each library.

Table S4 Significantly enriched GO term of DEGs in C28_F vs C28_M.

Table S5 DEGs between pseudomale formed under high temperature and normal temperature.

Table S6 Significantly enriched KEGG terms of DEGs down-regulated in C28_P vs C22_P.
