# Supplementary material for: Transcriptome of Gonads From High Temperature Induced Sex Reversal During Sex Determination and Differentiation in Chinese Tongue Sole, Cynoglossus semilaevis
Source: Front Genet. 2019 Nov 22;10:1128. doi: 10.3389/fgene.2019.01128 (PMC6882949; doi:10.3389/fgene.2019.01128)
Supplement: Table S1 — qRT-PCR primers used for validating RNA-seq data. [file Table_1.pdf]

Table S1. qRT-PCR primers used for validating RNA-seq data

Gene symbol	Gene ID	Forward primer (5'-3')	Reverse primer (5'-3')
figla	103397268	TGATCATGACAGCAGTGGGT	TTCTCCATCCTCTGGGACTG
foxl2	103378228	ATAATCCACGACAGCAACGC	ATGGCGATGAGAGCCACATA
wnt5b	103382784	ACATTATTGGAGCCCAGCCT	GAACTGGTACTGGCACTCCT
hspa13	103377660	AAGTGATGCAGCAGGAGAGT	TTTCAACAGCAGCCTGGAAC
hsc70-like	103378288	AATCATCGCCAACGACCAAG	CTGGTTCTTGGCTGCATCTC
hsd11b1-like	112488507	AGAGTCTCTCAGAGGAGCCA	ACAACCTGCTGTAACATCGC
hsd17b3-like	103398902	TATGTCCAGAAGCTGCCCAA	AGACACCAGTGGGAAGGATG
aromatase-like	103378914	TGCTACGTTGGAGCTGAAGA	CCGACATTGACGGATATGGC
cyp11b-like	103388284	CTCATCCTCCTGCCTGAGAG	ACAGCGTTGGTGAATATGGC
