# Supplementary material for: Transcriptome of Gonads From High Temperature Induced Sex Reversal During Sex Determination and Differentiation in Chinese Tongue Sole, Cynoglossus semilaevis
Source: Front Genet. 2019 Nov 22;10:1128. doi: 10.3389/fgene.2019.01128 (PMC6882949; doi:10.3389/fgene.2019.01128)
Supplement: Table S2 — Reads filtered information of each library. [file Table_2.pdf]

Table S2. Reads filtered information of each library

Sample	Total Raw Reads (M)	Total Clean Reads (M)	Total Clean Bases (Gb)	Clean Reads Q20 (%)	Clean Reads Q30 (%)	Clean Reads Ratio (%)
C22_M1	74.6	68.36	6.84	97.73	89.94	91.64
C22_M2	75.82	69.43	6.94	97.63	89.57	91.57
C22_M3	73.23	66.73	6.67	97.62	89.52	91.12
C22_F1	73.27	67.22	6.72	97.72	89.9	91.73
C22_F2	73.46	67.36	6.74	97.66	89.64	91.69
C22_F3	77.04	69.94	6.99	97.1	88.25	90.79
C22_P1	75.91	69.52	6.95	97.65	89.62	91.58
C22_P2	75.93	69.24	6.92	97.52	89.13	91.2
C22_P3	77.13	70.05	7.01	97.14	88.42	90.82
C28_M1	76.71	69.64	6.96	97.01	87.95	90.79
C28_M2	74.65	67.91	6.79	97.04	88.08	90.97
C28_M3	74.64	67.66	6.77	97	87.96	90.64
C28_F1	74.65	67.54	6.75	97.03	88.02	90.47
C28_F2	73.2	63.34	6.33	96.81	87.39	86.54
C28_F3	77.13	69.34	6.93	96.97	87.91	89.9
C28_P1	77.63	69.91	6.99	97.1	88.28	90.07
C28_P2	72.88	65.65	6.56	97	88	90.08
C28_P3	77.14	70.02	7	96.96	87.8	90.77
30dpf_F1	73.49	66.14	6.61	97.59	89.35	90.01
30dpf_F2	73.48	67.3	6.73	97.61	89.41	91.59
30dpf_F3	75.62	69.8	6.98	97.76	89.94	92.31
30dpf_M1	75.6	69.41	6.94	97.8	90.12	91.8
30dpf_M2	75.79	69.51	6.95	97.68	89.69	91.72
30dpf_M3	73.11	66.84	6.68	97.85	90.29	91.42
