# Supplementary material for: Transcriptome of Gonads From High Temperature Induced Sex Reversal During Sex Determination and Differentiation in Chinese Tongue Sole, Cynoglossus semilaevis
Source: Front Genet. 2019 Nov 22;10:1128. doi: 10.3389/fgene.2019.01128 (PMC6882949; doi:10.3389/fgene.2019.01128)
Supplement: Table S3 — Reads mapping information from each library. [file Table_3.pdf]

Table S3. Reads mapping information from each library

Sample	Total Clean Reads (M)	Total mapping genome ratio	Uniquely mapping genome ratio	Total mapping gene ratio	Uniquely mapping gene ratio
C22_M1	68.36	88.28	69.71	79.41	68.17
C22_M2	69.43	89.5	67.45	82.64	68.4
C22_M3	66.73	89.02	66.42	82.31	67.64
C22_F1	67.22	88.58	67.23	81.49	66.92
C22_F2	69.52	88.25	66.32	80.76	65.99
C22_F3	69.24	88.19	65.02	81.52	66.43
C22_P1	67.36	88.66	65.43	81.92	66.22
C22_P2	69.94	89.25	68.99	80.42	67.46
C22_P3	70.05	88.63	62.98	81.63	65.43
C28_M1	69.64	89.57	68.96	82.5	69.86
C28_M2	67.91	88.39	58.97	83.91	66.94
C28_M3	67.66	89.17	66.24	82.89	69.79
C28_F1	67.54	88.05	67.89	80.01	66.83
C28_F2	63.34	87.82	63.71	81.18	67.14
C28_F3	69.34	88.75	62.8	82.85	68.33
C28_P1	69.91	89.51	67.88	82.39	69.46
C28_P2	65.65	88.67	65.7	82.17	67.89
C28_P3	70.02	88.74	65.72	81.14	68.09
30dpf_F1	66.14	90.48	70.72	83.65	69.71
30dpf_F2	67.3	90.46	70.92	83.65	70.01
30dpf_F3	69.8	90.75	72.08	83.9	71.11
30dpf_M1	69.41	90.73	67.94	84.77	68.03
dpf30_M2	69.51	90.8	72.51	83.68	72.01
dpf30_M3	66.84	90.34	71.54	83.36	71
