# Supplementary material for: Transcriptome of Gonads From High Temperature Induced Sex Reversal During Sex Determination and Differentiation in Chinese Tongue Sole, Cynoglossus semilaevis
Source: Front Genet. 2019 Nov 22;10:1128. doi: 10.3389/fgene.2019.01128 (PMC6882949; doi:10.3389/fgene.2019.01128)
Supplement: Supplementary file 7 [file Image_1.pdf]

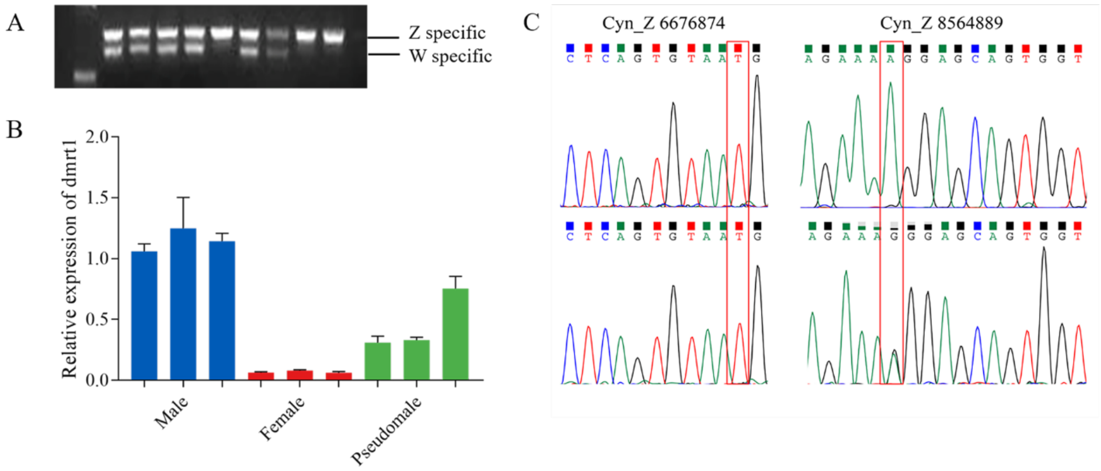


**Figure S1 Sex identification of *C. semilaevis*.**

(A). Genetic sex identification of *C. semilaevis* using sex linked SSR marker. (B). *dmrt1* relative expression level in male, female, and pseudomale. (C). Sex reversal associated SNP locus of Cyn_Z_6676874 and Cyn_Z_8564889 sequencing.
