# Supplementary material for: Transcriptome of Gonads From High Temperature Induced Sex Reversal During Sex Determination and Differentiation in Chinese Tongue Sole, Cynoglossus semilaevis
Source: Front Genet. 2019 Nov 22;10:1128. doi: 10.3389/fgene.2019.01128 (PMC6882949; doi:10.3389/fgene.2019.01128)
Supplement: Supplementary file 8 [file Image_2.pdf]

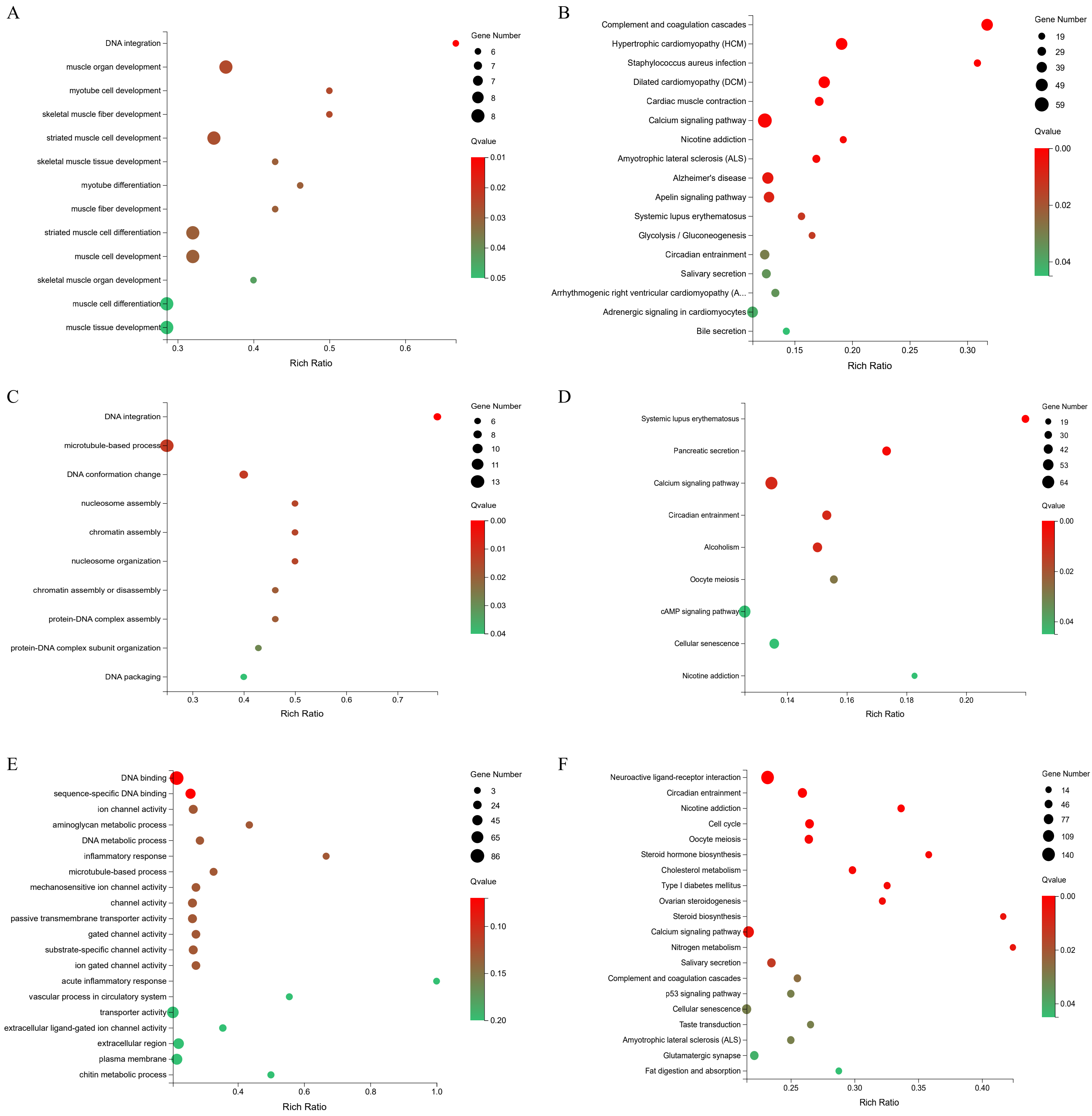

Figure S2 Analysis of DEGs between females and males

(A). Enriched GO terms of DEGs between 30dpf_F and 30dpf_M ($q < 0.05$). (B). Enriched KEGG terms of DEGs between 30dpf_F and 30dpf_M ($q < 0.05$). (C). Enriched GO terms of DEGs between C22_F and C22_M ($q < 0.05$). (D). Enriched KEGG terms of DEGs between C22_F and C22_M ($q < 0.05$). (E). Enriched GO terms of DEGs between C28_F and C28_M ($p < 0.05$, graph showing top 20 significant terms). (F). Enriched KEGG terms of DEGs between C28_F and C28_M ($q < 0.05$).
