# Supplementary material for: Transcriptome of Gonads From High Temperature Induced Sex Reversal During Sex Determination and Differentiation in Chinese Tongue Sole, Cynoglossus semilaevis
Source: Front Genet. 2019 Nov 22;10:1128. doi: 10.3389/fgene.2019.01128 (PMC6882949; doi:10.3389/fgene.2019.01128)
Supplement: Supplementary file 9 [file Image_3.pdf]

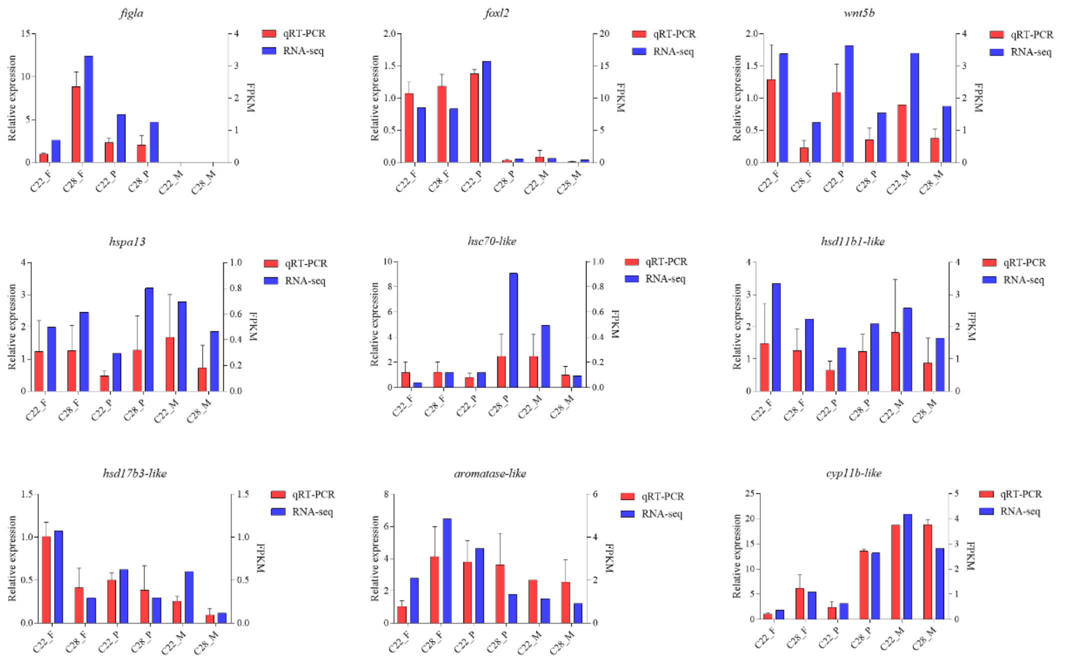


**Figure S3 qRT-PCR validation of RNA-Seq data**

Nine DEGs including *figla*, *foxl2*, *wnt5b*, *hspa13*, *hsc70-like*, *hsd11b1-like*, *hsd17b3-like*, *aromatase-like*, and *cyp11b-like* were selected for qRT-PCR. The left Y-axis represent the relative expression level of DEGs determined by qRT-PCR, and the right Y-axis represent FPKM determined by RNA-Seq. The scales of the left and right Y-axis are different. All data represent the mean value of three biological replicates. Error bars represent the standard errors of triplicates.
